# Therapeutic effects of long-circulating miR-135a-containing cationic immunoliposomes against gallbladder carcinoma

**DOI:** 10.1038/s41598-017-06234-8

**Published:** 2017-07-20

**Authors:** Guanghua Yang, Baobing Yin

**Affiliations:** 1 Department of General Surgery, Huashan Hospital Affiliated to Fudan University, Shanghai, China; 2 Department of General Surgery, Seventh People’s Hospital of Shanghai University of Traditional Chinese Medicine, Shanghai, China; 3 Biliary Disease Institute of Fudan University (proposed), Shanghai, China; 4 Department of General Surgery, Jing’an Branch of Huashan Hospital Affiliated to Fudan University (Jing’an District Centre Hospital of Shanghai), Shanghai, China

## Abstract

Gallbladder carcinoma (GBC) is the most common malignant tumour in the biliary tract, but effective therapeutics are lacking. Based on our previous studies, miR-135a is a potential tool to inhibit GBC proliferation. In this study, we constructed miR-135a-loaded DSPE-PEG2000 liposomes modified with Anti-EGFR antibodies (Anti-EGFR-CIL-miR-135a). The results of an analysis of their physicochemical properties indicated the particle size of it was 222.0 ± 2.1 nm in diameter with an uptake efficiency of 86.5%. Next, the post-treatment biological behaviours of GBC, specifically, invasion, metastasis and apoptosis, were evaluated. miR-135a inhibited GBC invasion and metastasis and promoted apoptosis compared to controls. Additionally, miR-135a targeted and regulated the expression of ROCK1, HOXA10 and BCL-2. Due to the targeted effects of Anti-EGFR-CIL-miR-135a, the GBC tumour growth rate was 60% lower in an *in vivo* xenograft-bearing mouse model compared to controls. Thus, Anti-EGFR-CIL-miR-135a is a promising therapeutic strategy to combat GBC.

## Introduction

Gallbladder carcinoma (GBC) is the most common malignant tumour of the biliary tract and comprises 80–95% of biliary tract malignancies. GBC prognosis is poor because traditional therapy is not effective. The 5-year survival rate is approximately 5% due to late-stage diagnoses^1, 2^. Therefore, a new and effective therapeutic strategy targeting GBC is urgently needed.

Recently, microRNAs (miRNAs) have become a hotspot in cancer treatment research^3, 4^ because they have shown excellent anticancer properties based on their ability to target multiple effectors in pathways involved in cell differentiation, proliferation and survival^5^, although a comprehensive mechanism remains elusive. Chandra *et al*. characterized GBC miRNAs, such as mir-34a, miR-335, miR-135-5p, miR-26a, miR-1, miR-145 and miR-146b-5p, as downregulated tumour-suppressive miRNAs. The exogenous expression of miR-1, miR-145 and miR-146b-5p in GBC cells induces apoptosis and blocks cell proliferation^6^. Given these effects, this strategy may be viable for the treatment of various cancers^7–9^.

In our previous studies, we analysed a miRNA microarray containing four paired GBC and para-cancerous tissues and observed 10-fold downregulation of miR-135a expression in GBC tissues, which was further confirmed in an expanded sample set. Exogenous miR-135a inhibited GBC cell proliferation by regulating the very low density lipoprotein receptor (VLDLR)-P38-mitogen-activated protein kinase (MAPK) axis^10^. In this study, we further explored the relationship between miR-135a and the pathogenic behaviours of GBC cells, including invasion, metastasis and apoptosis, to determine if miR-135a is an appropriate gene therapy target for GBC.

There are many challenges facing the targeted delivery of miRNAs, including limited stability in serum, rapid blood clearance, off-target effects, low bioavailability, nonspecific immune responses caused by low transfection efficiency, inferior tissue penetration, and vulnerability to nuclease degradation. Therefore, it is a prerequisite to identify an outstanding gene delivery vector to deliver miRNAs. Gene vectors consist of both viral and non-viral vectors. Liposomes, a type of non-viral vector, have been widely studied compared with other gene vectors. However, traditional liposomes are characterized by low cellular uptake, a short circulatory half-life, potential off-target effects and hypersensitivity reactions. Based on the high expression of epidermal growth factor receptor (EGFR) at the gallbladder cell membrane^11–13^, we constructed Anti-EGFR-CILs, assessed their physicochemical properties and active targeting, and further evaluated the antitumour effects of Anti-EGFR-CIL-miR-135a as a therapeutic strategy to treat GBC.

## Methods

### Ethical approval and informed consent

Gallbladder cancer and matched adjacent non-tumourous gallbladder tissues (2 cm away from the tumour) were obtained from surgical specimens from Huashan Hospital (Shanghai, China). Each case was diagnosed by pathological examination. Informed consent was obtained from each patient who provided tissue, and the research protocol was approved by the Ethics Committee of Huashan Hospital (ethical permit number: 2012-108). Animal use and care were in accordance with animal care guidelines that conformed to the Guide for the Care and Use of Laboratory Animals published by the US National Institutes of Health. All animal experiments were performed with the approval of the Shanghai Medical Experimental Animal Care Commission. Additionally, all procedures were conducted per the Helsinki Ethical Principles for medical research.

### Cell line, animal model and materials

A human gallbladder cancer cell line (GBC-SD) was purchased from the Institute of Cell Banks (Shanghai, China). Female nude mice (BALB/c mice, 5–6 weeks old and weighing 18–20 g) were purchased from the Chinese Academy of Sciences (SLAC, Shanghai, China). pWPXL as a kind of plamid backbone vector and pWPXL-miR-135a (miR-135a) were gifted by Dr. Didier Trono (School of the Life Sciences, Fudan University). An EGFR antibody was purchased from JingEn Biotech (Nanjing, China). PEG-2OSU, 1,2-dioleoyl-3-trimethyl-ammoniumpropane (DOTAP), distearoylphosphatidylcholine (DSPC) succinylphosphatidylethanolamine (DSPE-mPEG), and distearoyl-N-(3-carboxy-propionoylpoly (ethyleneglycol) succinl) phosphatidylethanolamine (DSPE-PEG-COOH) were purchased from Avanti Company (USA). N-hydroxysulfosuccinimide, 1-ethyl-3-(3-dime-thylaminopropyl) carbodiimide (EDC) and Chol were purchased from Sigma Aldrich (Shanghai, China). Cy5.5 was purchased from GE Healthcare (USA). NotI and NdeI were purchased from TAKARA (Japan). Protamine sulphate was purchased from Sangon Biotech (Shanghai, China). HilyMax transfection reagent was purchased from Dojindo Company (Kumamoto, Japan). DAPI and FITC-inulin were purchased from ROCHE Diagnostics GMBH (Germany) and Sigma Aldrich (USA). Cell Counting Kit-8 (CCK8) was purchased from Dojindo (Kumamoto, Japan). A caspase 3 assay kit was purchased from Keygentec Inc. (Shanghai, China). An ANNEXIN V-PI cell apoptosis assay kit was purchased from Life Technology (USA). FBS and RPMI 1640 medium were purchased from Gibco (USA).

### Liposome preparation

Long-circulating cationic liposomes (LCLs) were produced via the film-dispersion and hydration-sonication methods. DSPC, Chol, HOOC-PEG-DSPE/mPEG-DSPE and DOTAP were mixed in a 40:10:20:12.5 ratio and dissolved in chloroform. The mixture was transferred to a rotary evaporator for film dispersion at 150 rpm and 50 °C for one hour. miR-135a (110 ng/μl), FITC-inulin (100 ng/μl) or Cy5.5 (100 ng/μl) was added to the lipid film and then sonicated for 4 minutes at 150-watt power at 30 °C. Crude LCLs were loaded with miR-135a, FITC-inulin or Cy5.5 and then filtered using a 0.22-μm PC membrane (MILLEX GP Filter Unit, 0.1 μm, Merck Millipore Ltd., Germany). We adopted the protocol of Kirpotin *et al*. to measure liposome concentrations, and 6 mg/ml liposomes were added to monoclonal Anti-EGFR antibodies to generate complete long-circulating cationic immunoliposomes (Anti-EGFR-CILs)^14, 15^.

### Liposome characterization

#### Morphology, size distribution, and ζ-potential

Liposomes were loaded onto copper grid films for morphological observation via transmission electron microscopy (TEM) (Tecnai G2 20, China). Particle size distribution and surface charge (PBS, pH 7.4) were measured using a Nicomp 380/ZLS Zetasizer (Nano ZS, Malvern Instruments Ltd., UK).

#### Drug entrapment efficiency and drug loading efficiency

The particles of Anti-EGFR-CIL-miR-135a suspension was centrifuged via ultracentrifugation (4 °C, 45,000 rpm), and then plasmid extraction was performed. The concentration of pWPXL-miR-135a was measured by photometer detection at 260 nm in supernatant (Cz). The precipitate was added to 20 μl of 10% Triton X-100, and then plasmid extraction was performed. The concentration of miR-135a was measured via the same method (Ct). The entrapment efficiency was calculated as Ct/(Cz + Ct) * 100%^16^. The concentration of phospholipids in the liposomes was determined by performing ammonium ferrothiocyanate spectrophotometry at a wavelength of 488 nm. Drug loading efficiency was calculated by dividing the miR-135a concentration by the phospholipid concentration.

#### Controlled-release efficacy assay

The particles of Anti-EGFR-CIL-miR-135a suspension was sealed in phosphate-buffered saline (PBS) (pH = 7.4) with a 10 ml dialyser bag (3000 KD). Samples were collected every 24 hours for 15 days to measure the miR-135a plasmid concentration using a UV260 NanoDrop spectrophotometer compared to a normal control sample in which miR-135a was directly dissolved in PBS (pH = 7.4).

#### Restriction enzyme stability assay

We added 1 μl each of the restriction enzymes NotI and NdeI to 10 μl liquid individually containing miR-135a, LCL-miR-135a and Anti-EGFR-CIL-miR-135a at the concentration of plasmids being 100 ng/μl for 3 hours at 37 °C. After digestion, the plasmids were purified via the phenol-chloroform method, underwent agarose gel electrophoresis (140 V, 20 minutes) and were compared to normal controls.

### *In vitro* assays

#### Liposome cellular uptake efficiency assay

The cellular uptake of LCL-FITC and Anti-EGFR-CIL-FITC into GBC-SD cells was evaluated using a fluorescence microscope. GBC-SD cells were transfected with LCLs and Anti-EGFR-CILs containing FITC at a FITC-inulin concentration of 100 nmol/l.

#### Cytotoxicity and cell proliferation assays

The cytotoxic effects of empty liposomes (LCLs, Anti-EGFR-CILs) at concentrations ranging from 20 μg/ml to 500 μg/ml against cells were measured by performing a CCK-8 assay. Cell viability was calculated with the following formula:

$${([A}_{{\rm{D}}}-{{\rm{A}}}_{{\rm{B}}}{]/[A}_{{\rm{C}}}-{{\rm{A}}}_{{\rm{B}}}])\times \mathrm{100} \% $$

(A_D_: absorption after 24 and 48 hours of drug perturbation, A_C_: absorption of parallel untreated controls after 24 and 48 hours, A_B_: absorption of untreated groups at 0 hours). In addition, cell proliferation was measured by performing a CCK-8 assay after dividing the samples into five groups: Hilymax-miR-135a, LCL-miR-135a, Anti-EGFR-CIL-miR-135a, Anti-EGFR-CIL-pWPXL as a negative control individually at 100 ng/μl that was a concentration of plasmids inside the liposomes and a blank control.

#### Scratch wound assay

GBC-SD cells were seeded into 24-well plates at a density of 1 × 10^5^ cells per well and incubated overnight. The cells at the bottom of each well were then scratched using a 10-μl tip, and the floating cells were washed twice gently with medium. Afterwards, the cells were transfected with Anti-EGFR–CIL-pWPXL, Hilymax-miR-135a, LCL-miR-135a or Anti-EGFR-CIL-miR-135a. Finally, each well was imaged using a microscope to calculate the change in healing at 0 hours, 24 hours and 48 hours.

#### Transwell assay

GBC-SD cells were transfected with Anti-EGFR-CIL-pWPXL, Hilymax-miR-135a, LCL-miR-135a or Anti-EGFR-CIL-miR-135a. Twenty-four hours after transfection, the cells were seeded into Matrigel-plated upper wells (5 × 10^4^/well), and the lower wells contained 500 μl of complete medium, following a routine procedure. After incubation for 48 hours, each upper well was cleared using swabs, and the lower wells were measured by performing a CCK-8 assay for 2 hours at 37 °C. Detection at 450 nm with a BIO-TEK MQX200 Universal Microplate Reader (Bio-Tek, USA) was performed to evaluate cell migration ability.

#### Apoptosis and necrosis assays based on Annexin V-PI measurement

GBC-SD cells were seeded into 24-well plates at a density of 1 × 10^5^ cells per well and incubated overnight. The cells were then transfected with Anti-EGFR-CIL-pWPXL, Hilymax-miR-135a, LCL-miR-135a or Anti-EGFR-CIL-miR-135a. Forty-eight hours after transfection, cells in each well were digested and treated with 200 μl of binding buffer. Two microliters of Annexin V-FITC were added into each reaction, shaken gently and placed for 15 minutes at room temperature away from light. Four microliters of propidium iodide were added into the cell suspension. Apoptosis and necrosis were detected using a flow cytometry instrument (Attune NxT, USA).

### *In vivo* assays

#### Tumour burden model

A total of 5 × 10^6^ tumour cells were embedded in the left flanks of 5-week-old BALB/c mice via subcutaneous injection. After the tumours grew to 100 mm^3^ in volume, the tumor-bearing mice were divided into five groups (as mentioned above), and 1.5 mg/kg (plasmid to mouse body mass) was administered into the caudal vein. In addition, normal mice were divided into four groups: blank control, miR-135a, LCL-miR-135a, Anti-EGFR-CIL-miR-135a for analysing the distribution of miR-135a in the mice.

#### Inhibitory effects and distribution in the mice

Mice were administered a single dose of 30 μg of Anti-EGFR-CIL-miR-135a via tail-vein injections. This dose is equivalent to 1.5 mg/kg body weight, assuming that an average mouse weight is 20 g. Tumours were collected after 12 days of drug administration. Tumour volumes were measured as V (mm^3^) = (Major axis * Minor axis^2^)/2. The effects of Anti-EGFR-CIL-miR-135a on tumour size were compared to other groups. The anti-tumour rate (%) was calculated as follows:

$$\begin{array}{c}{(\mathrm{tumour\; volume\; of\; control\; group}(\mathrm{mm}}^{{\rm{3}}})-{\mathrm{tumour\; volume\; of\; treatment\; group}(\mathrm{mm}}^{{\rm{3}}})\\ /\mathrm{tumour\; volume\; of\; control\; group}({\mathrm{mm}}^{{\rm{3}}})\times \mathrm{100} \% {\rm{.}}\end{array}$$

In addition, the whole blood, liver, kidney, spleen, lung, heart and gallbladder of normal mice were collected 12 hours after injection and subjected to RNA isolation and quantitative reverse transcription PCR (qRT-PCR).

#### *In vivo* imaging

To observe the real-time distribution and tumour accumulation of fluorescent Cy5.5-loaded liposomes in BALB/c nude mice bearing GBC xenografts, whole-animal imaging was recorded using a Carestream FX Pro *in vivo* imaging system. Mice were administered single doses of 30 μg of Anti-EGFR-CIL-miR-135a via tail-vein injections. LCL-Cy5.5 or Anti-EGFR-CIL-Cy5.5 were administered via the tail vein. The mice were anaesthetized via an intraperitoneal injection of chloral hydrate and placed on an animal plate heated to 37 °C. Fluorescent scans were performed at various time points (1, 6 and 24 hours) post intravenous (i.v.) infection.

#### qRT-PCR

Total RNA from tumour and organ tissues was extracted using a Cell Culture and Tissue Total RNA Extraction and Preparation Mini Kit according to the manufacturer’s instruction. The quantity and quality of the RNA was confirmed with a NanoDrop 1000. Primers were designed using Primer Premier 5.0 software and synthesized by Generay Biotech Co, Ltd. Quantitative real-time PCR was performed using a KAPPA SYBR Green Supermix PCR kit and an iCycler apparatus system (Bio-Rad) (Table 1).

**Table 1:** Quantitative PCR primers sequence.

Gene	Forward (5′->3′)	Reverse (5′->3′)
miR-135a RT Primer	5′-CTCAACTGGTGTCGTGGAGTCGGCAATTCAGTTGAGTCACATAG-3′	
miR-135a	CCAGGCTTCCAGTACCATTAGG	GTTTCCGAGAGAGGCAGGTG
HOXA10	CAACTGGCTCACGGCAAAGA	GCTGCGGCTAATCTCTAGGC
BCL-2	GTGTGTGGAGAGCGTCAACC	GCATCCCAGCCTCCGTTATC
ROCK-1	GGAGAAGGAGGAGGAGATCAGTAA	TAGCAACACTTGTAGAATCCGAGAG
GAPDH	GAGTCCACTGGCGTCTTCAC	TGCTGATGATCTTGAGGCTGTT

Quantitative PCR primer sequences. U6 was used as an invariant housekeeping internal control gene for miR-135a, and GAPDH was used as an invariant housekeeping internal control gene for HOXA10, BCL-2, and ROCK-1.

#### Immunofluorescence

Tissues were fixed in 10% neutral buffered formalin solution and processed to 3-μm-thick paraffin sections. Immunohistochemistry was performed using a 3-step indirect process based on the labelled peroxidase complex method. Images were captured on a ZeissLSM510 Meta laser-scanning confocal microscope.

#### TUNEL assay

Tissues were fixed in 4% paraformaldehyde overnight and embedded in paraffin using a standard histological procedure. A terminal deoxynucleotidyl transferase-mediated dUTP-biotin nick-end labelling (TUNEL) was performed in accordance with the manufacturer’s protocols. TUNEL-positive cells were detected with an Olympus BX61 microscope under 400x magnification.

#### Haematoxylin-eosin (HE) staining

Representative sections (4 μm) of liver, kidney, spleen, lung, heart and gallbladder were cut and stained with HE and were then scanned with a Scanscope CS slide scanner (Aperio Technologies, Vista, CA) and visualized with ImageScope software (Aperio Technologies). The original objective lens magnification was 400x.

### Statistical analysis

The results are shown as the mean and standard deviation (SD). Data were analysed by performing Student’s t-tests (two-tailed) in GraphPad Prism 5. P values < 0.05 were considered statistically significant. *p < 0.05; **p < 0.01; ***p < 0.001; n.s. represents not significant (p > 0.05).

## Results

### Preparation and characterization of liposomes

**Figure 1:**
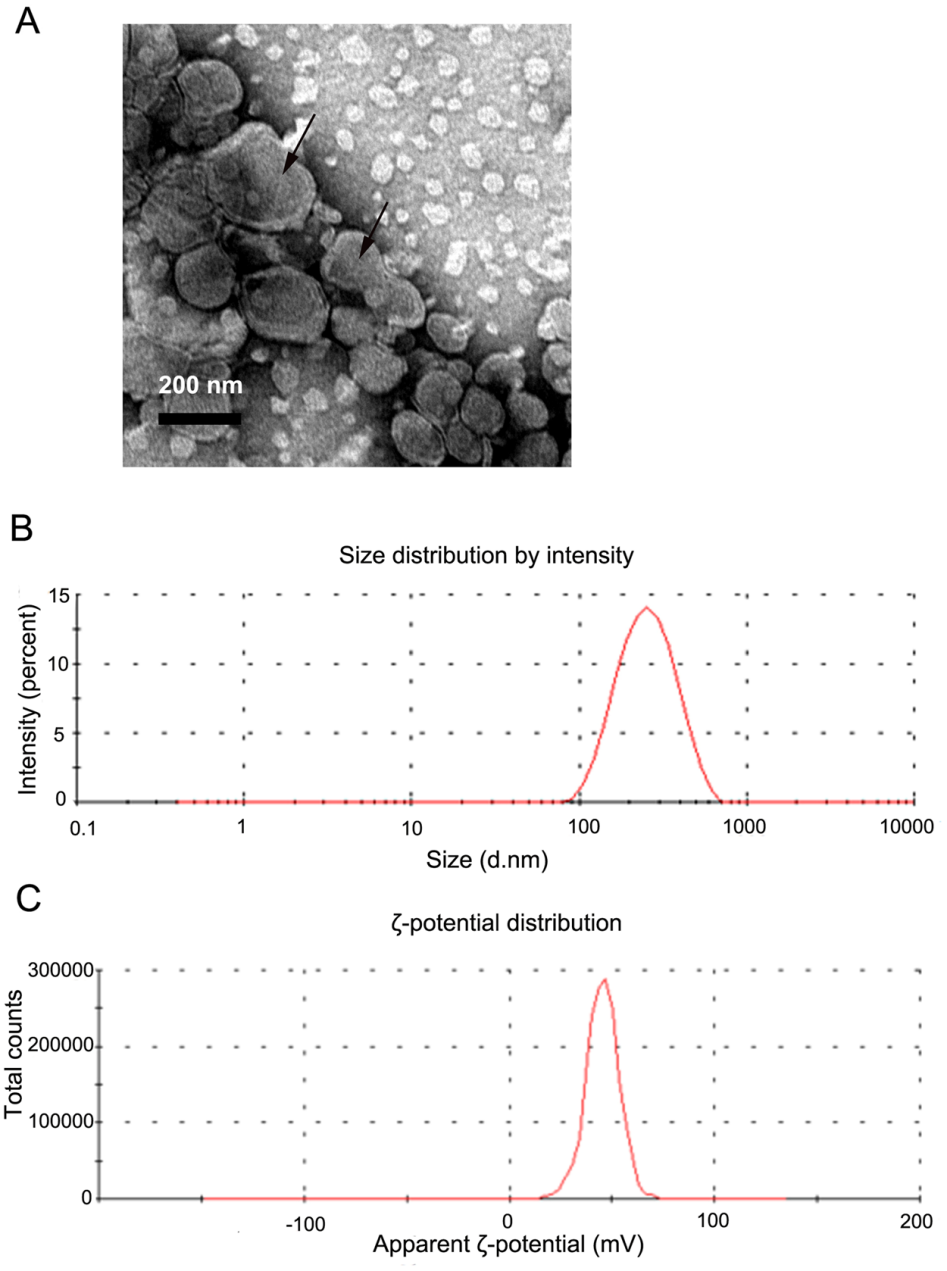
TEM photo showing 220-nm diameter liposomes and their distribution following phosphotungstic acid staining (**A**). The particle distribution (**B**) and ζ-potential (**C**) of Anti-EGFR-CIL-miR-135a.

The particle size of the Anti-EGFR-CIL-miR-135a was 222.0 ± 2.1 nm, with a polydispersity index (PDI) of 0.18 ± 0.02 and a positive ζ-potential of +42.93 ± 1.7 *mV* (Fig. 1B,C, Table 2). Based on TEM imaging, Anti-EGFR-CIL-miR-135a liposomes were spherical with a smooth surface (Fig. 1A). The encapsulation and drug loading efficiencies were 73.91% and 1.43%, respectively.

**Table 2:** C Characterization of the liposomes by size and ζ-Potential.

Liposomes	Size (d.nm)	Polydispersity (PdI)	ζ-Potential (mV)	Encapsulation efficiency (%)	Drug loading (%)
LCL-miR-135a	221.7 ± 4.4	0.16 ± 0.02	+30.43 ± 0.16	72.99 ± 0.15	1.42 ± 0.11
Anti-EGFR-CIL -miR-135a	222.0 ± 2.1	0.18 ± 0.02	+42.93 ± 1.7	73.91 ± 0.23	1.43 ± 0.26

ζ-potential, PDI, particle size, encapsulation efficiency, and loading efficiency of LCLs and Anti-EGFR-CILs. The data are expressed as the mean ± SD (n = 3) from three independent samples.

### Protective and controlled-release liposome efficacy

**Figure 2:**
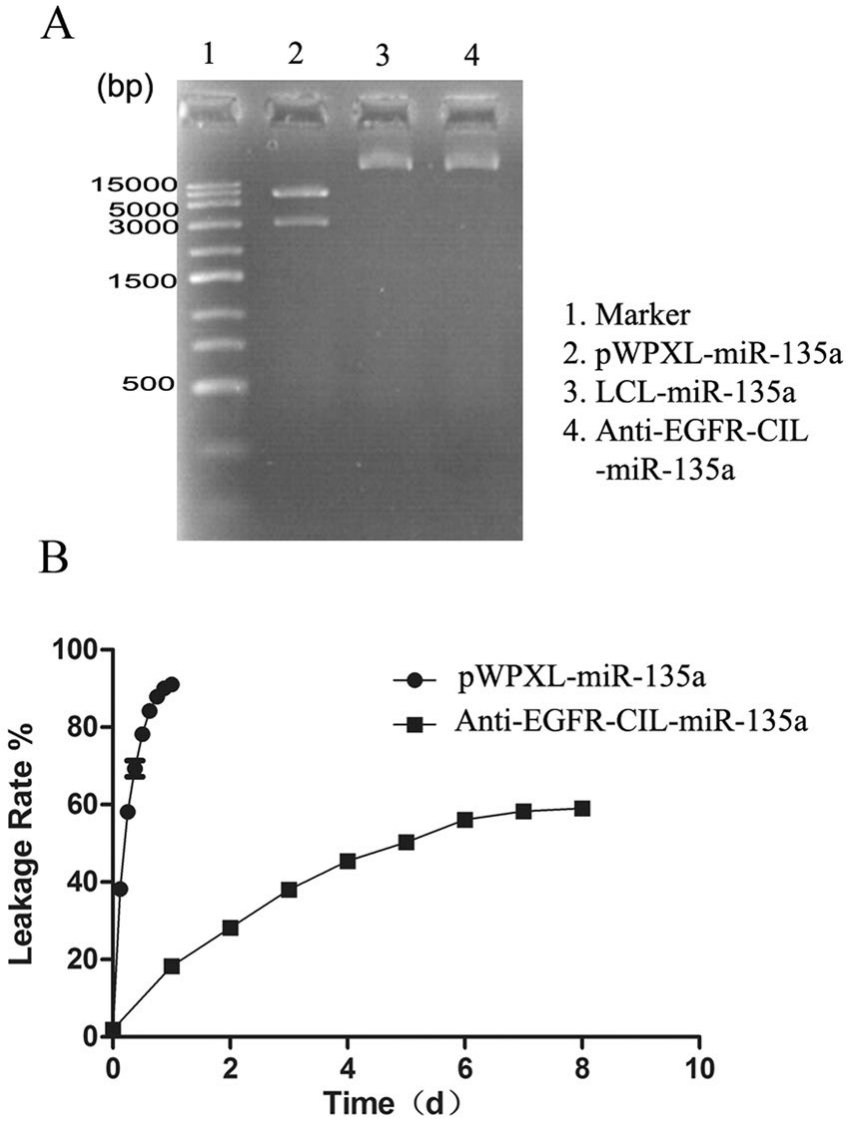
Plasmid enzyme experiments (**A**) and the controlled-release kinetics of Anti-EGFR-CIL-miR-135a and miR-135a in PBS buffer at 37 °C (**B**). The full-length gels from Fig. 2A are presented in Supplementary Figure 1.

LCLs and Anti-EGFR-CILs exerted strong protective effects on miR-135a according to plasmid enzyme experiments. Plasmids remained intact in the liposomes compared to non-encapsulated digested plasmids (Fig. 2A). The controlled-release kinetics of miR-135a from Anti-EGFR-CIL-miR-135a are shown in Fig. 2B. Approximately 50% of miR-135a from Anti-EGFR-CIL-miR-135a was released during the initial 15 days of dialysis. However, miR-135a alone exhibited a rapid release of 90% over 24 hours.

### Cellular uptake efficiency

**Figure 3:**
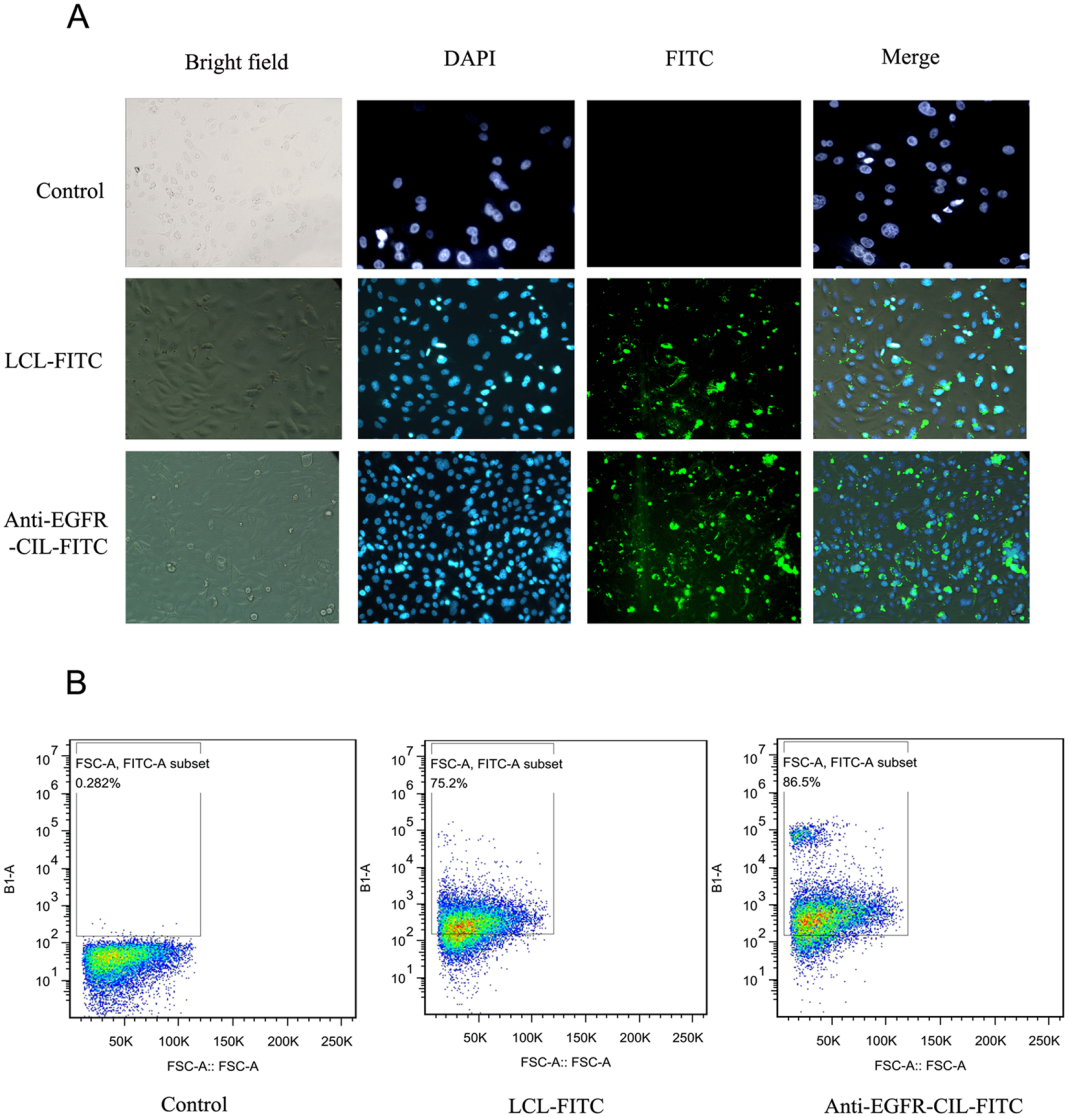
Fluorescence microscopy (**A**) and flow cytometry (**B**) images of GBC-SD cells incubated with various liposomes containing FITC at concentrations of 100 nmol/l for 24 hours.

The fluorescence intensity of GBC-SD cells treated with Anti-EGFR-CIL-FITC was far higher than that of cells treated with LCL-FITC (Fig. 3A). Additionally, flow cytometry data revealed a much stronger cellular uptake of Anti-EGFR-CIL-FITC than LCL-FITC (Fig. 3B). The *in vitro* cellular uptake efficiency was 86.5%.

### Cytotoxicity and cell proliferation assays

**Figure 4:**
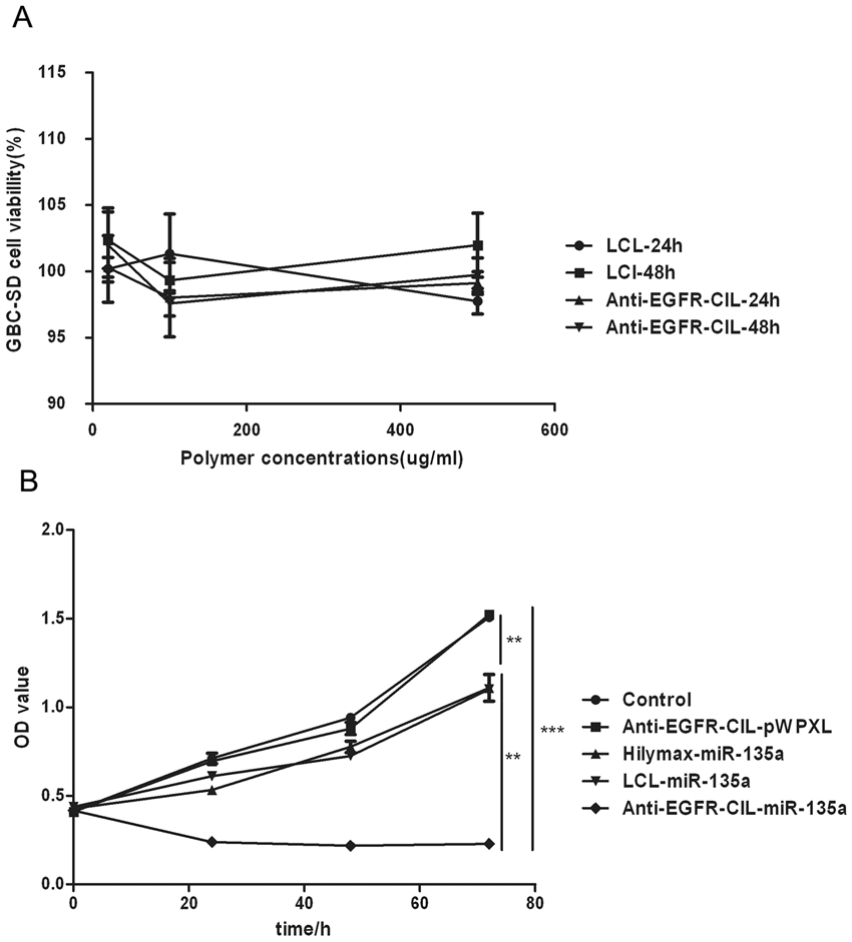
Liposome cytotoxicity. GBC-SD cells were incubated for 24 or 48 hours with varying concentrations of empty liposomes (**A**) and liposomes loaded with miR-135a at a concentration of 100 ng/μl for 24 hours (**B**). Cell viability was evaluated by performing a CCK-8 assay. Data (mean ± SD, n = 3) are representative of three independent experiments.

A cytotoxicity assay was performed to analyse the effects of empty liposomes and various liposomes loaded with miR-135a on cell viability. Empty liposomes exerted little toxicity on GBC-SD cells at concentrations of liposomes ranging from 20 μg/ml to 500 μg/ml. This low toxicity was reflected in the cell viability, which exceeded 90% even at the highest polymer concentration (Fig. 4A). Next, GBC-SD cells showed increased sensitivity to Hilymax-miR-135a, LCL-miR-135a and Anti-EGFR-CIL-miR-135a. Additionally, Anti-EGFR-CIL-miR-135a was the most toxic to GBC-SD cells compared with other treated groups (Fig. 4B).

### Cellular migration and Transwell assays

**Figure 5:**
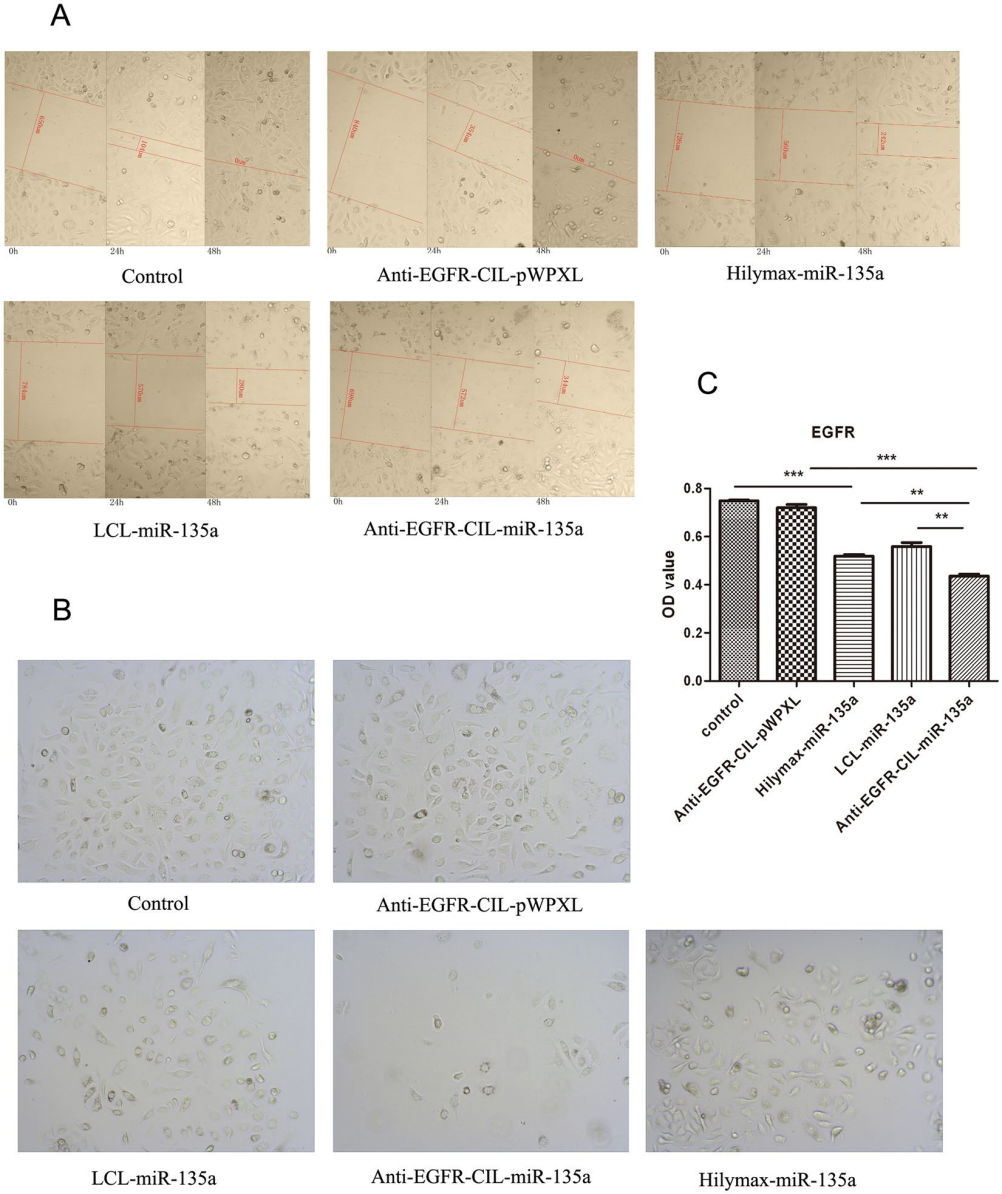
Scratch (**A**) and transwell (**B**) assays following Hilymax-miR-135a, LCL-miR-135a and Anti-EGFR-CIL-miR-135a treatment of GBC-SD cells at 0, 24 and 48 hours. (**C**) Invasive cell numbers of the Anti-EGFR-CIL-miR-135a group (LCL-miR-135a vs. Anti-EGFR-CIL-miR-135a; **p < 0.01).

Tumour cellular migration promotes the recurrence and progression of tumour diseases and thus is an important factor for tumour growth. In our experiments, complete wound closure was observed in the control group after 24 hours of incubation. However, larger wound areas remained uncovered following culture with Hilymax-miR-135a compared to the control group, suggesting pWPXL-miR-135a effectively blocked GBC-SD cellular migration. In addition, Anti-EGFR-CIL-miR-135a exerted strong inhibitory effects on migration compared with the other groups, and similar invasion results were observed in the transwell assay (Fig. 5A–C).

### Cellular apoptosis assays

**Figure 6:**
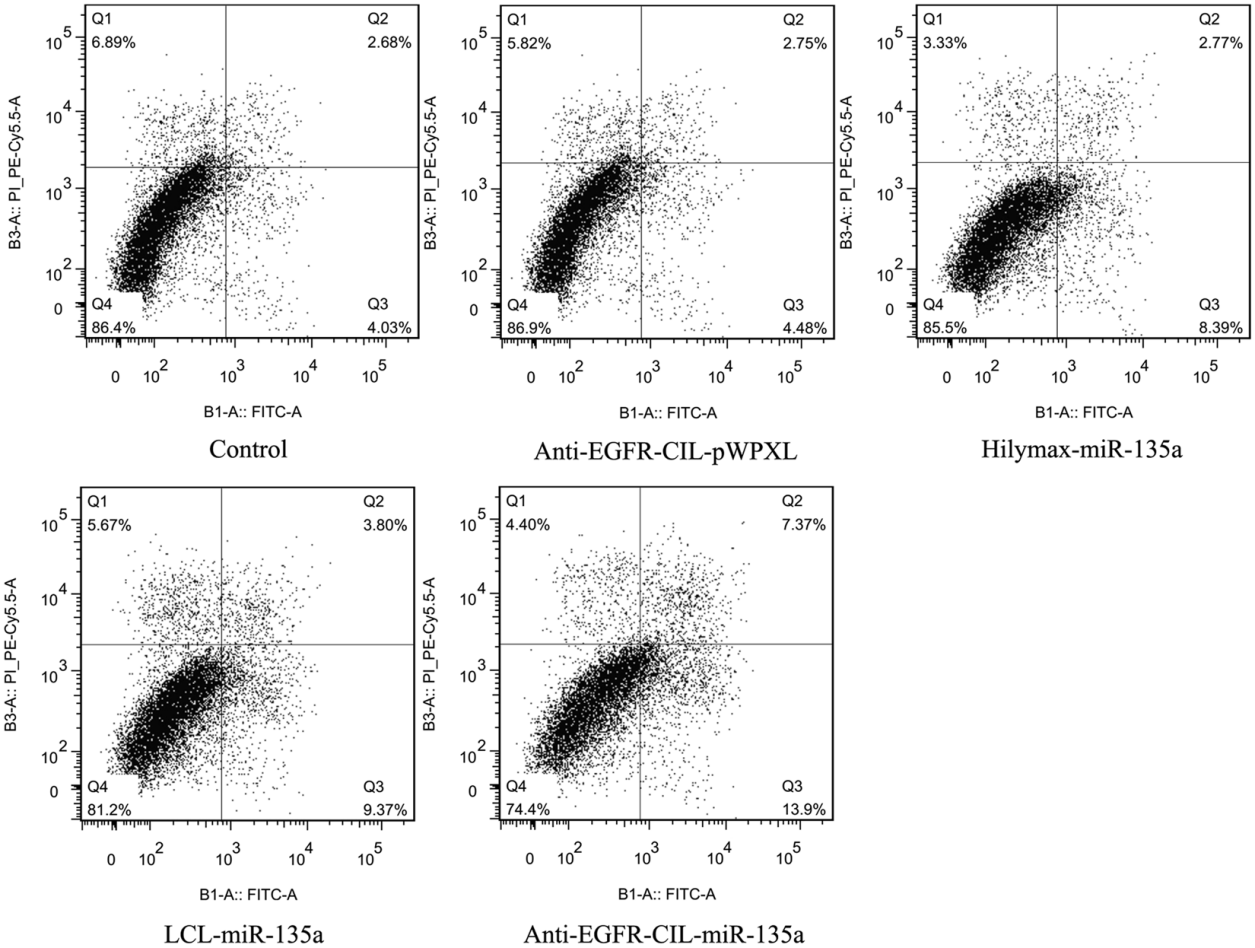
Annexin V/PI assays following Hilymax-miR-135a, LCL-miR-135a and Anti-EGFR-CIL-miR-135a treatment of GBC-SD cells at 24 hours. Data (mean ± SD, n = 3) are representative of three independent experiments.

To study the apoptotic effects of pWPXL-miR-135a on GBC-SD cells, Annexin V/PI assays were carried out. The apoptotic cell population percentage with Hilymax-miR-135a exceeded 11% (sum of Q2 and Q3) compared with the untreated control group at 6% (sum of Q2 and Q3). The Anti-EGFR-CIL-miR-135a group most strongly promoted GBC-SD cell apoptosis compared with the other groups (Fig. 6).

### *In vivo* biodistribution of delivered Anti-EGFR-CIL-miR-135a

**Figure 7:**
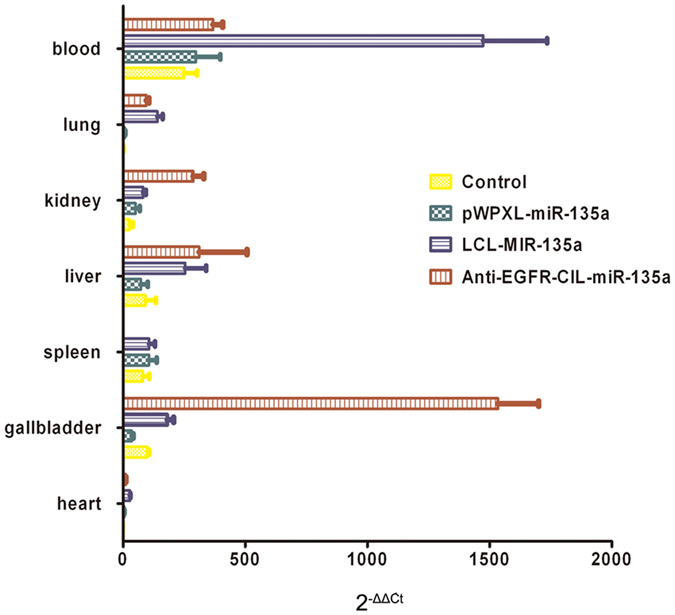
Biodistribution of systemically delivered miRNA- miR-135a. Six mice were injected each with 30 μg of naked miR-135a, LCL-miR-135a, or Anti-EGFR-CIL-miR-135a. After killing at 12 hours post injection, total RNA was isolated from whole blood, liver, kidney, spleen, lung, heart and gallbladder.

We first investigated the accumulation of Anti-EGFR-CIL-miR-135a into the gallbladder and other tissues 12 hours post-i.v. tail-vein injection. Increased miR-135a levels were detectable in all tissues tested in each groups. However, miR-135a did not appear to undergo specific distribution in the pWPXL-miR-135a group. The livers did not yield the highest miR-135a levels but the whole blood in the LCL-miR-135a group, which was unanticipated and differed from previous reports in which cationic lipid particles preferentially accumulated in the liver. Interestingly, miR-135a levels were higher in the gallbladders than in the other tissues, including the whole blood in the Anti-EGFR-CIL-miR-135a group (Fig. 7).

### *In vivo* imaging of delivered Anti-EGFR-CIL-miR-135a

**Figure 8:**
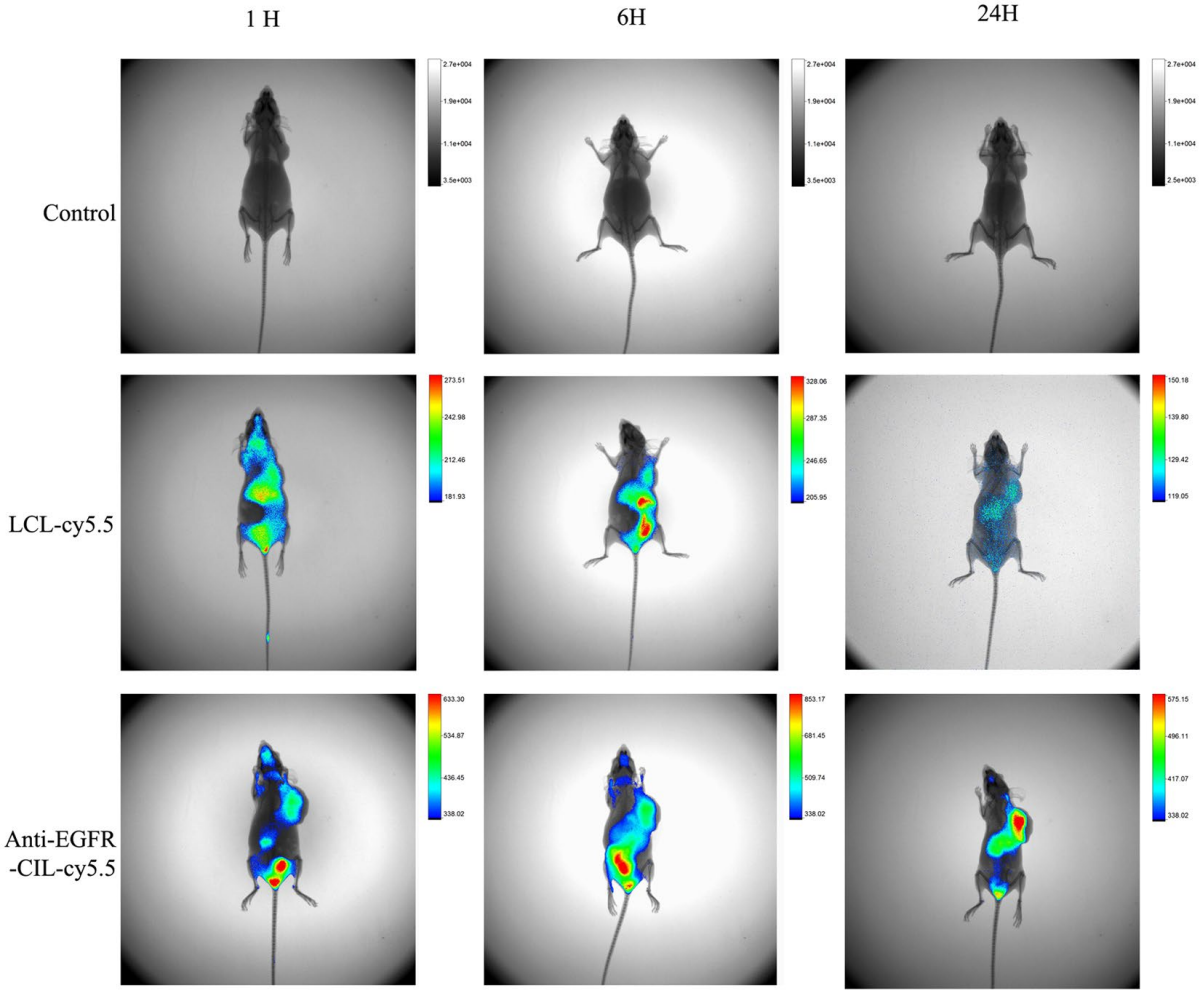
LCL-miR-Cy5.5 and Anti-EGFR-CIL-Cy5.5 were injected through the tail vein at a dose of 1.5 mg/kg mouse weight. After 30 minutes and 1, 6, and 24 hours post-injection, mice were sacrificed, and tumour tissues were analysed using an imaging system.

The tumour targeting efficiency of liposomes in GBC-SD cancer cell-bearing mice was determined using an *in vivo* fluorescence imaging system. Images were taken at 1, 6, and 24 hours post-injection. Over time, fluorescence intensity in the tumours of mice treated with Anti-EGFR-CIL-Cy5.5 was significantly higher than that of mice treated with LCL-miR-Cy5.5, and fluorescence gradually concentrated at tumour sites in the 24-hour group (Fig. 8).

### Antitumour efficiency

**Figure 9:**
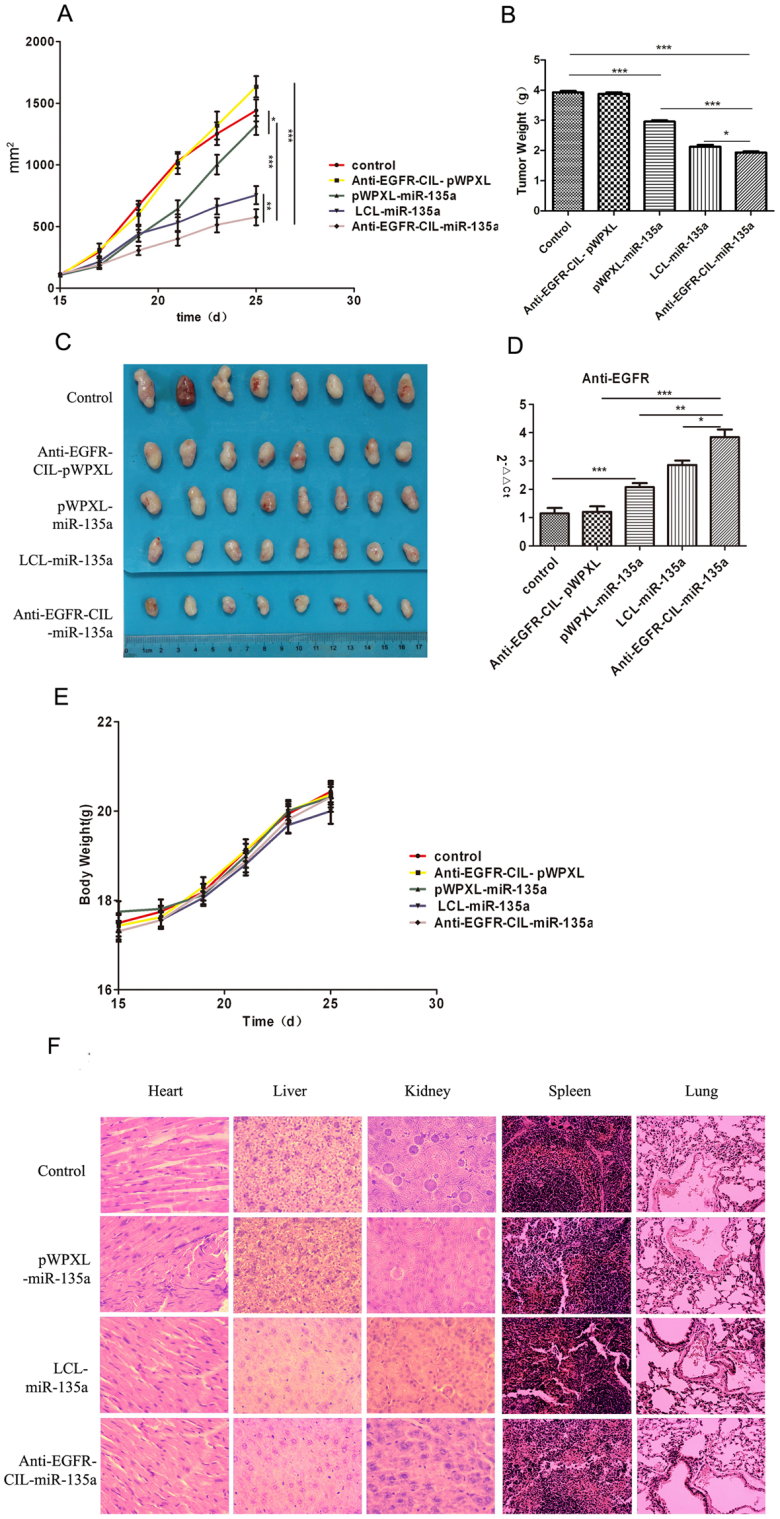
The therapeutic effects of liposomes in mice bearing subcutaneous GBC tumours. Mice were treated with i.v. injections via the tail vein of LCL-miR-135a, Anti-EGFR -CIL-miR-135a or naked miR-135a (1.5 mg/kg). Therapy was given once on days 15, 17, 19, 21, 23 and 25 after inoculation. (**A**) The tumour growth curve. (**B**) The tumour weight curve. (**C**) Images of excised tumours from each group at the endpoint. On day 27, forty mice were euthanized, and tumours were excised. The effects of the drugs on GBC tumours *in vivo* were evaluated by determining tumour sphere formation. (**D**) miR-135a expression in the Anti-EGFR-CIL-miR-135a-treated group compared with expression in the other groups as determined by qRT-PCR. Data are expressed as the mean ± SD (n = 8). (**E**) The mouse weight curve. (**F**) HE staining of the viscera (*p < 0.05; **p < 0.01; ***p < 0.001).

The *in vivo* antitumour efficiency of Anti-EGFR-CIL-miR-135a was assessed in GBC-SD cancer-bearing mice. Significant decreases in tumour size were clearly observed among the mice that received pWPXL-miR-135a, LCL-miR-135a, and Anti-EGFR-CIL-miR-135a compared with blank control mice. This decreasing trend significantly changed with increased therapy time. Moreover, the tumour growth inhibition of mice treated with Anti-EGFR-CIL-miR-135a was much higher than that of mice treated with miR-135a and LCL-miR-135a. Its anti-tumour rate of Anti-EGFR-CIL-miR-135a was 60% (Table 3) at 12 days post-injection. The sizes and weights of the tumours in the Anti-EGFR-CIL-miR-135a group were obviously reduced in comparison to the control (p < 0.001), miR-135a (p < 0.001) or LCL-miR-135a groups (p < 0.01) (Fig. 9A–C). Similarly, miR-135a levels in the Anti-EGFR-CIL-miR-135a group were higher than those in other groups as determined by qRT-PCR (Fig. 9D), confirming the effective antitumour efficiency of Anti-EGFR-CIL-miR-135a for GBC-SD cancer-bearing mice. In addition, the weights of the mice in each group were not obviously different (Fig. 9E). HE staining indicated no significant tissue damage and inflammation in mice treated with LCL-miR-135a and Anti-EGFR-CIL-miR-135a (Fig. 9F).

**Table 3:** The volume of tumor and anti- tumor rate.

Group	Volume (mm^3^)						The anti-tumor rate of 25d (%)
	15d	17d	19d	21d	23d	25d	
Control	107.99 ± 16	295.82 ± 40	670.61 ± 104	1031.86 ± 165	1252.45 ± 259	1441.46 ± 249	—
Anti-EGFR- CIL-pWPXL	103.74 ± 32	310.13 ± 148.13	598.15 ± 235	1014.98 ± 254	1319.15 ± 319.16	1634.39 ± 243	−0.13
pWPXL-miR- 135a	105.6 ± 17	179.9 ± 72.05	423.98 ± 137.02	643.6 ± 195.36	1002.03 ± 225	1320.35 ± 217	0.08
LCL-miR- 135a	105.94 ± 21	213.84 ± 80	440.28 ± 176	531.18 ± 189.59	661.97 ± 180	754.68 ± 209	0.48
Anti-EGFR- CIL-miR-135a	117.18 ± 23	187.28 ± 60.31	305.5 ± 107	400.16 ± 155	512.9 ± 171	574.97 ± 184	0.60

Tumour volumes in mice bearing GBC xenografts on days 15, 17, 19, 21, 23, and 25 after inoculation and anti-tumour rates on day 25 after inoculation. Data are expressed as the mean ± SD (n = 8).

### TUNEL assay

**Figure 10:**
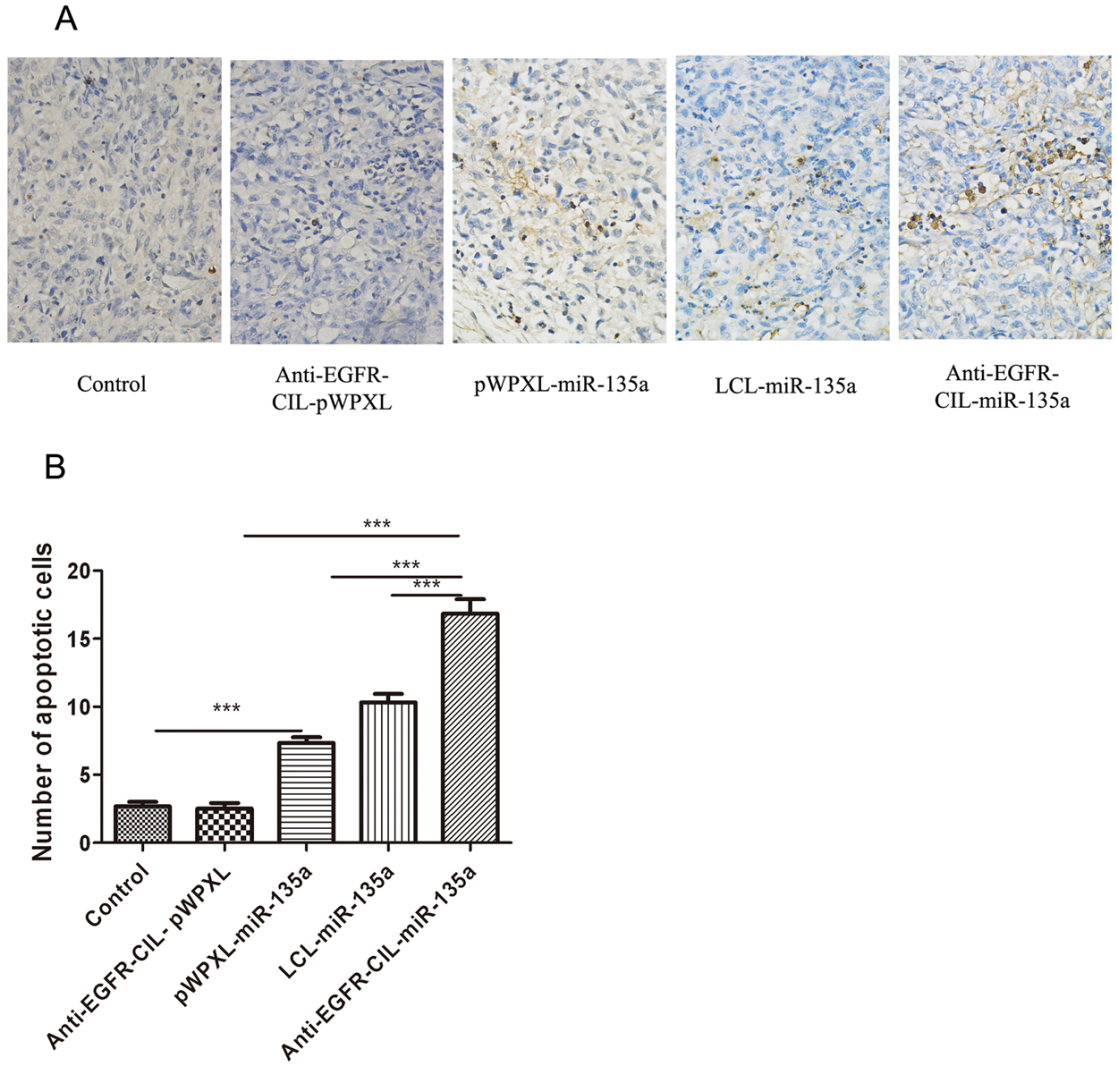
TUNEL assay results for apoptotic cells in GBC-SD cancer (**A**). Quantitative data were shown in (**B**). Data were represented as the mean ± SD (n = 5) (*p < 0.05; **p < 0.01; ***p < 0.001).

TUNEL assay results further indicated apoptosis caused by Anti-EGFR-CIL-miR-135a treatment was increased in comparison to that resulting from either pWPXL-miR-135a (p < 0.001) or LCL-miR-135a treatment (p < 0.01). Thus, high levels of apoptosis are an important factor for tumour inhibition (Fig. 10A,B).

### Downregulation of BCL-2, HOXA10 and Rock1 expression by Anti-EGFR-CIL-miR-135a *in vivo*

**Figure 11:**
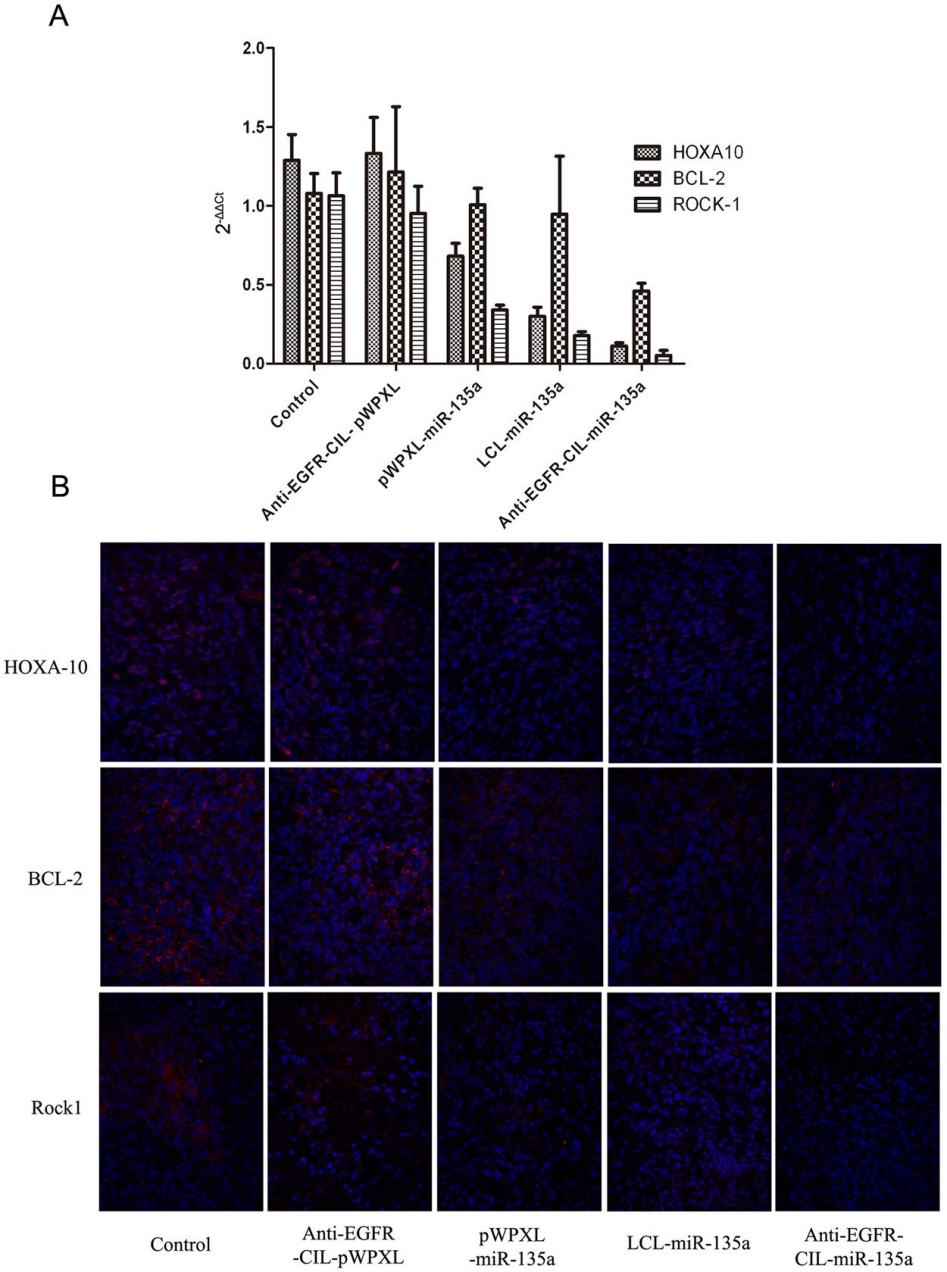
The Anti-EGFR-CIL-miR-135a downregulated the expression of target genes *in vivo*. (**A**) Down-regulated BCL-2, HOXA10 and Rock1 expression were assessed at the mRNA level by performing qRT-PCR and (**B**) at the protein level by performing an immunofluorescence assay 24 hours after mice were sacrificed and analysed. miR-135a, LCL-miR-135a and Anti-EGFR-CIL-miR-135a at a miR-135a concentration of 1.5 mg/kg (*p < 0.05 and **p < 0.01) (n = 8).

Expression of the predicted target genes BCL-2, HOXA10 and Rock1 was efficiently downregulated at both the mRNA and protein levels in GBC-SD cancer-bearing mice treated with pWPXL-miR-135a compared with control mice (p < 0.01). In addition, BCL-2, HOXA10 and Rock1 expression in the Anti-EGFR-CIL-miR-135a group was downregulated to the greatest degree compared to expression in the other groups (p < 0.05) (Fig. 11A,B).

## Discussion

Successful tumour-targeting gene therapy should effectively interfere with various biological tumour behaviours, including proliferation, invasion, and metastasis, and should promote apoptosis. miR-135a has been reported to influence the biological behaviours of many tumours, for example, by inhibiting growth in renal cell carcinoma and pancreatic ductal adenocarcinoma by targeting c-MYC and Bmi1^17, 18^, inhibiting invasion and metastasis in early gastric cancer by downregulating ROCK1^19^, and increasing apoptosis and improving disease-free survival in lymphoma^20^. Based on this evidence, we sought to manipulate miR-135a in GBC therapy research.

According to our previous studies, exogenous miR-135a expression inhibited the proliferation of GBC cells by regulating the VLDLR-P38-MAPK axis. In our current study, miR-135a significantly inhibited GBC-SD invasion and metastasis and clearly promoted apoptosis. In addition, using the PicTar, TargetScan, and miRBase databases, we predicted the target genes of miR-135a in GBC to be ROCK1, HOXA10 and BCL-2, which are associated with the migration, invasion and apoptosis of various tumours^21, 22^. The miR-135a reduces the invasion and metastasis of prostate cancer by inhibiting ROCK1^23^ and induces the apoptosis of various cells by inhibiting HOXA10 and BCL-2 expression^24–26^. Based on this evidence, our studies preliminarily indicated that ROCK1, HOXA10 and BCL-2 expression were down-regulated following the transfection of exogenous miR-135a in mice bearing GBC xenografts. Notably, this study only examined the relationship between miR-135a and the biological behaviours of GBC cells, specifically invasion, metastasis and apoptosis. Our preliminary studies regarding related regulatory mechanisms must be further confirmed.

An ideal gene delivery system, including its degradation products, should have a long circulatory half-life *in vivo*, active or passive targeting abilities, an efficient uptake rate, low toxicity and no immunogenicity^27^. Liposomes are a type of non-viral vector that have been widely studied compared with other gene vectors. However, single typological liposomes, such as ordinary liposomes and cationic liposomes, present low cellular absorption capacity, short circulatory half-lives, potential off-target effects and hypersensitivity reactions. Therefore, we created CILs with decreased side effects that achieved the desired effects, such as precise targeting, good biological compatibility, efficient gene transfection ability and a longer circulation time *in vitro* and *in vivo* ^28^.

The reasonable structures and excellent physicochemical properties of liposomes make them ideal gene carriers. Liposomes containing DSPE-PEG2000 provide surface-exposed hydrophilic PEG moieties, which prevent liposome aggregation, making them more stable while reducing their interactions with blood proteins, opsonins, antibodies, and enzymes and avoiding phagocytosis and reticuloendothelial system identification to persistently extend the amount of time a drug is in circulation^29–32^. Similarly, DSPC and cholesterol play similar roles in liposome composition. In addition, we conjugated ligands with the carboxyl of DSPE-PEG2000-COOH. This not only retained the long circulatory characteristics of the PEGylation cationic liposomes but also avoided the high mobility PEG chain, inhibiting the coupling reaction between the active groups on the surface of the liposomes and ligands^33^. We analysed drug distribution in the body and found that the blood concentration of miR-135a in the Anti-EGFR-CIL-miR-135a group was higher than that in the pWPXL-miR-135a group 24 hours after drug injection, indicating the characteristic long life cycle of Anti-EGFR-CILs. In addition, miR-135a expression in the Anti-EGFR-CIL-miR-135a group was highest in the gallbladder, followed by the liver, 24 hours after the injection of Anti-EGFR-CIL-miR-135a. Normally, liposomes that traverse through the endothelial gap are easily taken up by the liver and spleen, but based on our experimental data, Anti-EGFR-CILs in nude mice exhibited a strong tendency to migrate to the gallbladder. This may be attributable to the high expression of EGFR in the gallbladder cells of nude mice. In addition, plasmid enzyme digestion and release rate experiments indicated the strong protective effects and sustained release properties of Anti-EGFR-CILs.

Reasonable particle size and surface charge are important factors for passive targeting and the combinatorial abilities of liposomes. Anti-EGFR-CILs had a smooth surface and quasi-circular average size of 222 nm. Liposomes with sizes ranging between 30 nm and 300 nm not only effectively reduce the first-pass elimination effect but also effectively extravasate and aggregate in tumour tissues due to their enhanced permeability and retention (EPR)^27^. In addition, the PDI (0.17) and **ζ**-potential (+47 *mV*) of Anti-EGFR-CILs were ideal as determined by performing TEM and analysis with a nanoparticle analyser, indicating superior transfection and encapsulation abilities because of cationic affinity to cell membrane and DNA-Liposome charge interaction^34, 35^. Finally, according to a toxicity test, LCLs and Anti-EGFR-CILs with doses of liposomes ranging from 20–500 ng/μl, liposomes were safe for cells, exhibiting good biocompatibility and no cytotoxicity.

Reasonable ligand targets are the most important factor affecting the active targeting ability of liposomes^36, 37^, not only increasing the transfection rate of cationic liposomes *in vitro* but also compensating for the disadvantage of the low transfection rate of cationic liposomes caused by gene release ahead of time and cationic liposome aggregation *in vivo* ^38^. During treatment of a variety of solid tumours, particularly digestive system tumours, carriers modified by Anti-EGFR have shown obvious synergistic attenuation and antitumour effects^39–41^. Therefore, we fabricated Anti-EGFR-CILs loaded with miR-135a based on the high EGFR expression at the GBC cell membrane^11–13^. The encapsulation rate of Anti-EGFR-CILs was 73.91%, the drug loading rate was 1.43%, and the cellular uptake efficiency was 86.5% *in vitro*. The encapsulation efficiency and drug loading rate did not differ between Anti-EGFR-CILs and LCLs, while the transfection efficiency of Anti-EGFR-CILs was significantly higher than that of LCLs. In addition, the Anti-EGFR-CIL-Cy5.5 group showed the strongest fluorescence compared with the other groups *in vivo* imaging. Similarly, miR-135a content in the Anti-EGFR-CIL-miR-135a group was also highest by qRT-PCR. Finally, according to our trend analysis of tumour volume changes, the anti-tumour effects of the Anti-EGFR-CIL-miR-135a group were strongest, with an inhibitory rate of 60%, which was higher than that of the other groups. Based on the above data, Anti-EGFR-CILs demonstrated possessed more specificity to actively target GBC compared with other control groups^42–44^, gathering at the tumour tissue surface to transfect greater amounts of miR-135a plasmid into the tumour tissue^45–47^. In summary, Anti-EGFR-CILs are a safe and efficient delivery system, and miR-135a exerts strong therapeutic effects on GBC. Thus, the Anti-EGFR-CIL-miR-135a represents a promising therapeutic strategy against GBC.

## Electronic supplementary material

Supplementary information
