# Supplementary material for: Therapeutic effects of long-circulating miR-135a-containing cationic immunoliposomes against gallbladder carcinoma
Source: Sci Rep. 2017 Jul 20;7:5982. doi: 10.1038/s41598-017-06234-8 (PMC5519676; doi:10.1038/s41598-017-06234-8)
Supplement: Supplementary file 1 — Supplementary information [file 41598_2017_6234_MOESM1_ESM.pdf]

Therapeutic effects of long-circulating miR-135a-containing cationic immunoliposomes against gallbladder carcinoma

Guanghua Yang, Baobing Yin

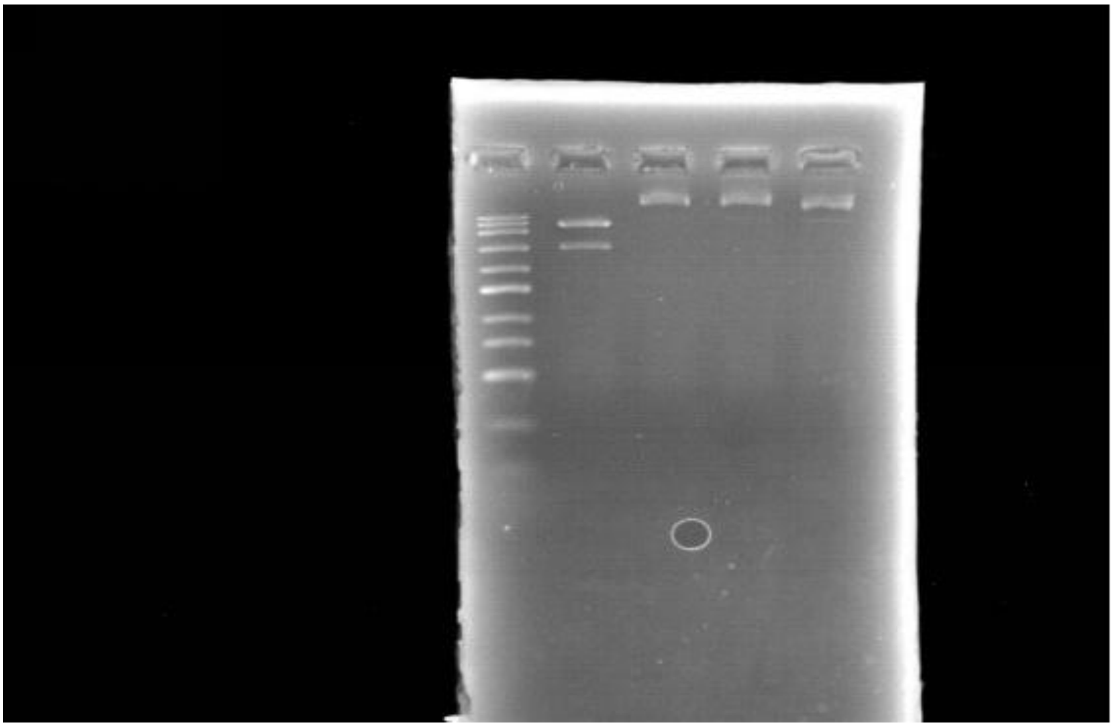

Supplementary figure 1. The full-length gels of Figure 2A
